# Infective Endocarditis Complicated With Septic Embolic Stroke After Transcatheter Aortic Valve Implantation: A Case Report

**DOI:** 10.7759/cureus.25379

**Published:** 2022-05-26

**Authors:** Suchapa Arayakarnkul, Koravich Lorlowhakarn, Sarinya Puwanant, Suphot Srimahachota, Aekarach Ariyachaipanich

**Affiliations:** 1 Internal Medicine, Chulalongkorn University, Bangkok, THA; 2 Cardiovascular Medicine, Chulalongkorn University, Bangkok, THA; 3 Cardiology, King Chulalongkorn Memorial Hospital, Bangkok, THA

**Keywords:** tavi, tavr, transcatheter aortic valve replacement, transcatheter aortic valve implantation, stroke, prosthetic valve endocarditis, infective endocarditis

## Abstract

Transcatheter aortic valve implantation (TAVI) is a relatively novel procedure developed for aortic stenosis (AS) management in patients with moderate to high surgical risk, especially the elderly with multiple comorbidities. Infective endocarditis following transcatheter aortic valve implantation (post-TAVI-IE) is an uncommon complication that contributes to very high morbidity and mortality. Further complications from post-TAVI-IE include ischemic stroke from septic emboli. Here, we report a case of an 82-year-old man with severe symptomatic AS who underwent TAVI, presenting with fever and alteration of consciousness, which was diagnosed as post-TAVI-IE per Duke criteria complicated by acute hemiparesis from septic emboli stroke. He was treated successfully conservatively using antibiotics. We have reviewed the options of treatment and outcomes for post-TAVI-IE.

## Introduction

Aortic stenosis (AS) is a slowly progressive condition with increasing prevalence with advancing age and is one of the most commonly encountered valvular diseases worldwide. Once it produces symptoms such as dyspnea, chest pain, or heart failure, it is already in advanced stages. Transcatheter aortic valve replacement (TAVI) has been developed as a novel intervention to target elderly patients who hold moderate to high risks [1] to undergo surgical aortic valve replacement (SAVR) in severe stages, as defined by reduced valve area and high flow gradient across the aortic valve, and symptomatic AS patients with high risk for surgical intervention whose life expectancy is greater than one year [2].

Due to its less invasive nature, this fragile group of patients had a better chance of post-intervention recovery. Typical post-TAVI complications include permanent pacemaker implantation due to postoperative high-grade atrioventricular block, paravalvular regurgitation, and infection including infective endocarditis [3]. Infective endocarditis following transcatheter aortic valve implantation (post-TAVI-IE) is a serious complication with very high morbidity and mortality rates; 80% of the patients meet the criteria for surgical intervention and are reported to have a 75% mortality rate in one year [2,4]. This itself is a paradox since patients initially selected for TAVI procedures in the first place are the group that was previously deemed unfit for surgery. Therefore, despite a high proportion of patients having met the indication for surgical intervention, most cases were managed conservatively. Data on the mortality rates between surgical and conservative treatment have suggested the survival rates to be comparable [4,5], but the data is still very limited. There is still a gap in knowledge regarding the most appropriate course of action in the management of TAVI-IE.

Here, we report a case of an 82-year-old man with severe symptomatic AS who underwent TAVI, presenting with fever and alteration of consciousness, and was diagnosed as post-TAVI-IE per Duke criteria complicated by acute hemiparesis from a septic embolic stroke.

## Case presentation

An 82-year-old patient with severe AS and ureteral stricture with nephrostomy who recently underwent TAVI presented with fever and alteration of consciousness. He had a history of fever for the past week, as confirmed by the body temperature of 38.3°C at presentation. Physical examination was notable for a diastolic murmur and valve clicks at the right parasternal border. No splinter hemorrhage, Janeway lesions, or Osler nodes were noted.

Three months prior to this presentation, the patient was diagnosed and treated for non-ST elevation acute coronary syndrome and severe calcific AS with acute heart failure. Due to high operative mortality risk as assessed with the Society of Thoracic Surgeons (STS) score of 9.7% (cut-off value for high risk = 8%), percutaneous coronary intervention (PCI) and TAVI were performed instead of open surgery.

During EvolutR #34 mm (Medtronic, Minneapolis, Minnesota) valve deployment, his electrocardiogram turned to a complete atrioventricular block, necessitating a permanent pacemaker insertion. Post-implantation ventriculography showed a well-seated aortic prosthesis with mild paravalvular leakage at 7 o’clock with no residual pressure gradient between the aorta and the left ventricle. The left ventricular ejection fraction was increased from 48% to 64%, and the patient was discharged.

During this subsequent admission, the initial workup for the source of fever revealed marked leukocytosis (16,170/μL, neutrophil 87.8%) and numerous white blood cells on urinalysis. Therefore, he was initially treated for urinary tract infection. On the third day of admission, the patient developed a sudden-onset right hemiparesis. Brain computed tomography confirmed an acute infarction in the left parietal area. A transesophageal echocardiogram (TEE) showed a 6 mm x 10 mm mobile mass at the anterior mitral leaflet with mitral regurgitation due to leaflet perforation, as shown in Video 1.

**Video 1:** Transesophageal echocardiogram showing vegetation at anterior mitral leaflet with mitral regurgitation due to leaflet perforation

Infectious workup reported *Enterococcus faecalis* in both blood and urine cultures. Having met two major Duke criteria for IE, the patient was diagnosed with infective endocarditis after transcatheter aortic valve implantation (TAVI-IE) with septic embolic stroke. Considering septic emboli stroke complicating IE, our patient is indicated for surgery; nevertheless, taking into account the multiple comorbidities and high surgical risks, conservative treatment was decided upon. Antibiotics were switched to ampicillin and ceftriaxone. Following two weeks of intravenous antibiotics, he developed a new onset of fever. Blood culture was positive for* Pseudomonas aeruginosa*, which was thought to have been sourced from his nephrostomy. Intravenous ciprofloxacin was added to the regimen. A follow-up transthoracic echocardiogram (TTE) was done following four weeks of antibiotics, which showed a decreased size of the mitral valve vegetation at 4 mm x 8 mm. There is a newly seen 7-mm ventricular septal defect with left-to-right intracardiac shunt as shown as a high-velocity flow in the right ventricular chamber during systolic phase shown in Video 2.

**Video 2:** Transthoracic echocardiogram showing ventricular septal defect with left-to-right intracardiac shunt after infective endocarditis resolution

All three antibiotics continued for the duration of six weeks. In the following year, the patient returned to the hospital due to episodes of pseudomonal septicemia, all of which were managed conservatively with six weeks of intravenous antibiotics. The source of infection was suspected to be his nephrostomy, which the multidisciplinary team meeting had recommended removal. To date, two years after TAVI, the patient is still alive and did not develop any additional complications.

## Discussion

This case demonstrates post-TAVI-IE in a severe AS patient who underwent TAVI and later developed septic embolic stroke as a further complication. Post-TAVI-IE [6] itself is described to affect 0.3%-1.2% of all patients who underwent the procedure [7]. Male gender (proposed to be owing to poor dental and urologic hygiene), chronic kidney disease (due to frequent intravascular manipulation from hemodialysis), and a new pacemaker implantation (as an entry route for pathogen and endocardial lesion during insertion procedure) are found to be associated with post-TAVI-IE.

Diagnosis of post-TAVI-IE utilizes the modified Duke criteria [8]. The most common presentations are fever and heart failure [5], usually presenting within one-year post-procedure. The median time is at 5.4 months. Most frequently isolated causative organisms are gram-positive bacteria such as streptococcus (25.3%), staphylococci (25.3%), and enterococci (24.1%), with *Enterococcus *spp. as a leading cause in patients who had undergone the transfemoral catheter route [9]. The all-cause mortality of post-TAVI-IE has been reported to be as considerable at 75% one year after diagnosis [2].

Post-TAVI-IE may lead to various complications such as structural destruction (valvular regurgitation or ventricular septal defect), uncontrolled infection, or stroke. Risk factors for septic embolic stroke after TAVI include previous stroke (either at baseline or periprocedural TAVI stroke), residual aortic regurgitation more than or equal to moderate after TAVI, balloon-expandable valves, infective endocarditis within 30 days after TAVI, and vegetation size of more than 8 mm, especially those involving mitral valve. Incidence of stroke was reported to be at 6%-60% among those who underwent the procedure, with a higher probability in those with more risk factors. Mortality rates for this patient group were reported at 66% at one year [6].

Management of post-TAVI-IE and the following septic embolic stroke is a challenging medical dilemma. Persistent infection, uncontrolled embolism, or refractory heart failure may indicate an open-heart surgery [5,9], which was initially avoided in this group of patients who are deemed at high risk to undergo an operation. Therefore, despite having met indications for open-heart surgery, most patients were managed conservatively with antibiotics [10]. Previous literature on mortality rates comparing conservative and surgical management is still inconclusive. Some studies suggested the mortality rates are of no significant difference [4,5], while a meta-analysis reported a logistic regression model showing surgical treatment with self-expandable devices to relate to lower mortality [9]. Proposed management regarding post-TAVI-IE is shown in Table 1 with advantages and disadvantages of each modality, which include surgical intervention, conservative management, and very rarely, a TAVI-in-TAVI regarding post-TAVI-IE management [11].

**Table 1:** A literature review of infective endocarditis following transcatheter aortic valve implantation (post-TAVI-IE) management

Methods	Advantages	Disadvantages
Open cardiac surgery [10,12]	Eliminate the source of infection and correct structural abnormalities (i.e., dehiscence, paravalvular abscess, or fistula)	High perioperative risk, extensive length of stay, and expense
Conservative treatment (antibiotics) [12]	Noninvasive, comparable outcomes with surgical intervention	Probability of infection relapse and incapable of structural damage management
Valve-in-valve procedure [7]	Less invasive than open surgery, correction of aortic regurgitation	Very limited data, reported to be performed only after infection resolution

## Conclusions

Post-TAVI-IE is a serious complication following TAVI. This not uncommon complication leads to extensive morbidity and mortality. To date, no consensus management has been concerted. Since this patient population is very vulnerable to decompensation, the best effort should go toward its prevention. Nevertheless, in the event that post-TAVI-IE ensues, the most appropriate course of action varies, and individualized strategy should be discussed among the involved cardiovascular personnel and multidisciplinary team as deemed most fit to each patient’s distinctive setting.
